# Supplementary figures and images for: Clinical and laboratory factors associated with neonatal sepsis mortality at a major Vietnamese children’s hospital
Source: PLOS Glob Public Health. 2022 Sep 2;2(9):e0000875. doi: 10.1371/journal.pgph.0000875 (PMC10021837; doi:10.1371/journal.pgph.0000875)

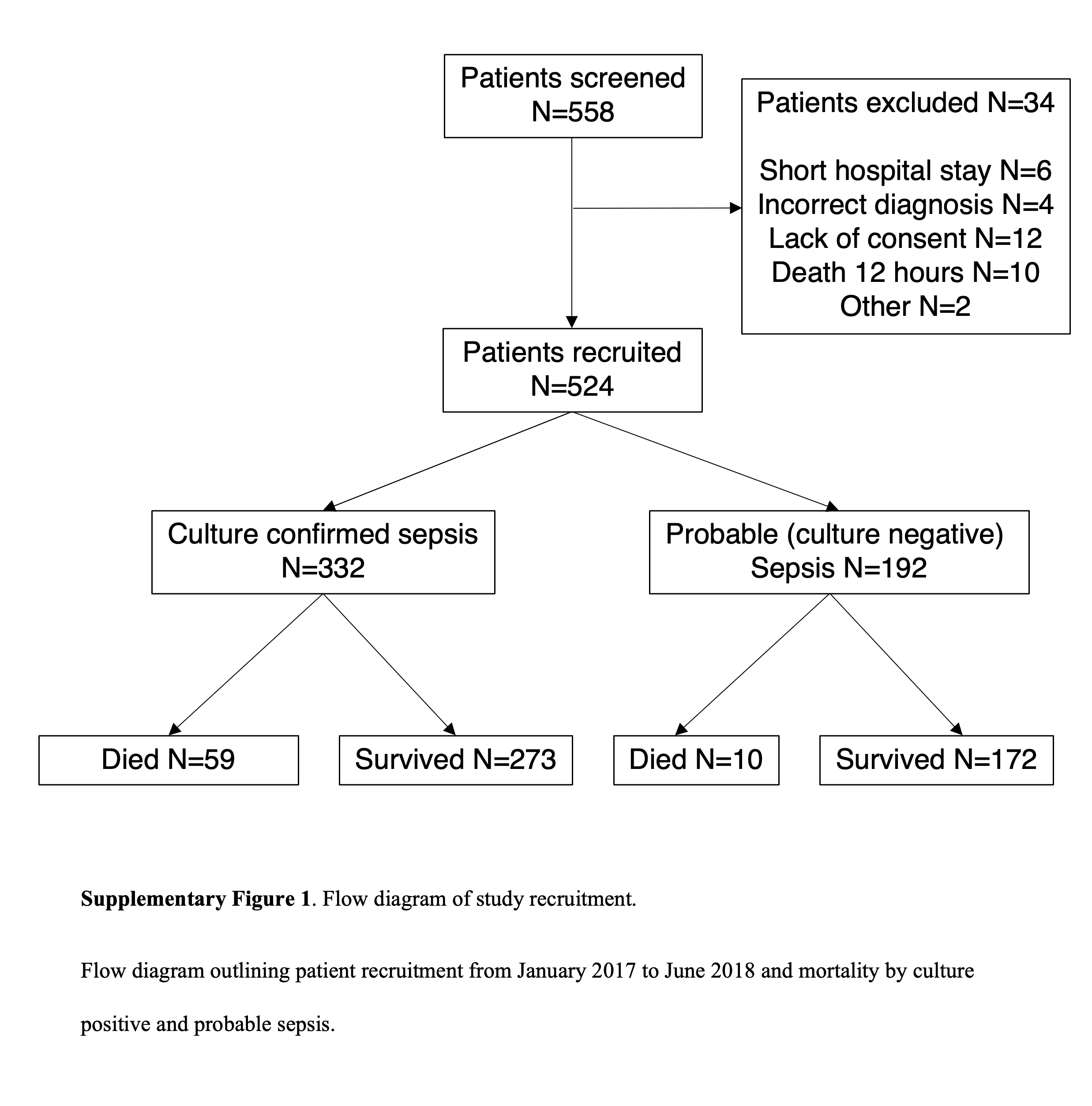

Supplement: S1 Fig — Flow diagram outlining patient recruitment from January 2017 to June 2018 and mortality by culture positive and probable sepsis. (TIF) [file pgph.0000875.s001.tif]
