# Supplementary material for: Clinical and laboratory factors associated with neonatal sepsis mortality at a major Vietnamese children’s hospital
Source: PLOS Glob Public Health. 2022 Sep 2;2(9):e0000875. doi: 10.1371/journal.pgph.0000875 (PMC10021837; doi:10.1371/journal.pgph.0000875)
Supplement: S1 Table — (DOCX) [file pgph.0000875.s002.docx]

**S1 Table.** Univariable analysis for factors associated with mortality in neonatal sepsis.

| **Characteristics** | **Died  (n = 69)** | | **Survived  (n = 455)** | | ***p*-values*** |
| --- | --- | --- | --- | --- | --- |
| **Demographic features** |  |  |  |  |  |
| Extreme prematurity | 12 | (17.4) | 17 | (3.7) | **<0.001** |
| Extremely low birth weight | 10 | (14.5) | 17 | (3.7) | **<0.001** |
| **Maternal features** |  |  |  |  |  |
| C-section delivery | 23 | (33.3) | 167 | (36.7) | 0.890 |
| Perinatal asphyxia | 2 | (2.9) | 12 | (2.6) | 0.900 |
| Intrapartum fever >38^o^C | 1 | (1.4) | 3 | (0.7) | 0.482 |
| Chorioamnionitis | 1 | (1.4) | 12 | (2.6) | 0.554 |
| Rupture of membranes >18 hours | 3 | (4.3) | 5 | (1.1) | **0.040** |
| **Neonatal sepsis classification** |  |  |  |  |  |
| Culture-confirmed sepsis | 59 | (85.5) | 273 | (60) | **<0.001** |
| Late-onset sepsis | 61 | (88.4) | 418 | (91.9) | 0.339 |
| Hospital-acquired sepsis | 67 | (97.1) | 317 | (69.7) | **<0.001** |
| **Clinical manifestations** |  |  |  |  |  |
| Fever >38.5^o^C | 12 | (17.4) | 160 | (35.2) | **0.003** |
| Hypothermia <36^o^C | 30 | (43.5) | 65 | (14.3) | **<0.001** |
| Temperature instability | 26 | (37.7) | 36 | (7.9) | **<0.001** |
| Bradycardia | 8 | (11.6) | 17 | (3.7) | **0.004** |
| Tachycardia | 5 | (7.2) | 7 | (1.5) | **0.003** |
| Rhythm instability | 3 | (4.3) | 5 | (1.1) | **0.040** |
| Reduced urinary output | 10 | (14.5) | 1 | (0.2) | **<0.001** |
| Hypotension | 46 | (66.7) | 27 | (5.9) | **<0.001** |
| Mottled skin | 44 | (63.8) | 40 | (8.8) | **<0.001** |
| Impaired peripheral perfusion | 45 | (65.2) | 41 | (9.0) | **<0.001** |
| Apnoea episodes | 33 | (47.8) | 54 | (11.9) | **<0.001** |
| Bradypnea | 2 | (2.9) | 3 | (0.7) | 0.075 |
| Tachypnoea | 1 | (1.4) | 8 | (1.8) | 0.854 |
| Increased oxygen requirements | 67 | (97.1) | 280 | (61.5) | **<0.001** |
| Mechanical ventilation | 62 | (89.9) | 129 | (28.4) | **<0.001** |
| Feeding intolerance | 60 | (87.0) | 189 | (41.5) | **<0.001** |
| Poor sucking | 58 | (84.1) | 234 | (51.4) | **<0.001** |
| Abdominal distension | 42 | (60.9) | 138 | (30.3) | **<0.001** |
| Petechial rash | 13 | (18.8) | 5 | (1.1) | **<0.001** |
| Sclerema | 16 | (23.2) | 2 | (0.4) | **<0.001** |
| Irritability | 0 | (0) | 4 | (0.9) | 1.000 |
| Lethargy | 24 | (34.8) | 48 | (10.5) | **<0.001** |
| Hypotonia | 20 | (29.0) | 21 | (4.6) | **<0.001** |
| Seizure | 3 | (4.3) | 13 | (2.9) | 0.502 |
| Severe jaundice | 24 | (34.8) | 112 | (24.6) | 0.073 |
| **Laboratory findings** |  |  |  |  |  |
| Leukopenia <4,000/mm^3^ | 16 | (23.2) | 13 | (2.9) | **<0.001** |
| Leucocytosis >20,000/mm^3^ | 33 | (47.8) | 173 | (38.0) | 0.120 |
| Immature to total neutrophil ratio >0.2 | 2 | (2.9) | 3 | (0.7) | 0.075 |
| Thrombocytopenia <100,000/mm^3^ | 43 | (62.3) | 92 | (20.2) | **<0.001** |
| C-reactive protein >15 mg/L | 51 | (73.9) | 268 | (58.9) | **0.017** |
| Hypoglycaemia <45 mg/dL | 15 | (21.7) | 40 | (8.8) | **0.001** |
| Hyperglycaemia >180 mg/dL | 21 | (30.4) | 34 | (7.5) | **<0.001** |
| Base excess < –20 mEq/L | 21 | (30.4) | 16 | (3.5) | **<0.001** |
| Serum lactate >4 mmol/L | 14 | (20.3) | 19 | (4.2) | **<0.001** |
| Total bilirubin (µmol/L) | 166.8 | (103.8–225.2) | 173.8 | (127.0–224.7) | 0.416 |
| Creatinine (µmol/L) | 63.8 | (42.2–125.6) | 53.1 | (40.6–72.9) | **0.002** |
| Alanine aminotransferase (IU/L) | 13 | (8–35) | 14 | (9–23) | 0.744 |
| Electrolyte disturbance | 51 | (73.9) | 113 | (24.8) | **<0.001** |
| Abnormal coagulation | 23 | (33.3) | 20 | (4.4) | **<0.001** |
| **Infection conditions/syndromes** |  |  |  |  |  |
| Pneumonia | 51 | (73.9) | 259 | (56.9) | **0.007** |
| Catheter-related bloodstream infection | 29 | (42.0) | 80 | (17.6) | **<0.001** |
| Necrotizing enterocolitis | 14 | (20.3) | 88 | (19.3) | 0.853 |
| Meningitis | 14 | (20.3) | 84 | (18.5) | 0.717 |
| Peritonitis | 10 | (14.5) | 19 | (4.2) | **<0.001** |
| Urinary infection | 2 | (2.9) | 4 | (0.9) | 0.142 |
| **Comorbidities** |  |  |  |  |  |
| Congenital heart diseases | 34 | (49.3) | 97 | (21.3) | **<0.001** |
| Congenital gastrointestinal anomalies | 18 | (26.1) | 80 | (17.6) | 0.091 |
| Respiratory distress syndrome | 13 | (18.8) | 45 | (9.9) | **0.027** |
| Bronchopulmonary dysplasia | 10 | (14.5) | 15 | (3.3) | **<0.001** |
| Pulmonary hypertension | 9 | (13.0) | 14 | (3.1) | **<0.001** |
| Congenital renal anomalies | 3 | (4.3) | 4 | (0.9) | **0.019** |
| **Treatment** |  |  |  |  |  |
| Duration of mechanical ventilation (days) | 8 | (3–21) | 4 | (2–7) | **<0.001** |
| Duration of parenteral nutrition (days) | 14 | (7–40) | 11 | (6–19) | **0.013** |
| Duration of lipid infusion (days) | 14 | (5–38) | 19 | (12–23) | 0.830 |
| Duration of central catheters (days) | 21 | (11–21) | 21 | (7–21) | 0.255 |
| Surgical/invasive intervention | 37 | (53.6) | 134 | (29.5) | **<0.001** |
| Shock management | 55 | (79.7) | 35 | (7.7) | **<0.001** |
| Blood products transfusion | 57 | (82.6) | 170 | (37.4) | **<0.001** |
| Antacids | 9 | (13.0) | 100 | (22.0) | 0.088 |
| Corticosteroids | 4 | (5.8) | 9 | (2.0) | 0.057 |
| **Severity and duration of stay** |  |  |  |  |  |
| Score of severity (NTISS) | 34 | (25–40) | 15 | (10–24) | **<0.001** |
| Severe sepsis | 56 | (81.2) | 65 | (14.3) | **<0.001** |
| Septic shock | 46 | (66.7) | 27 | (5.9) | **<0.001** |
| Duration of stay (days) | 29 | (8–64) | 23 | (13–38) | 0.365 |

Values are n (%) or median (IQR), *****Wilcoxon rank-sum test, Chi-squared test or Fisher’s exact test
