# Supplementary material for: Clinical and laboratory factors associated with neonatal sepsis mortality at a major Vietnamese children’s hospital
Source: PLOS Glob Public Health. 2022 Sep 2;2(9):e0000875. doi: 10.1371/journal.pgph.0000875 (PMC10021837; doi:10.1371/journal.pgph.0000875)
Supplement: S2 Table — (DOCX) [file pgph.0000875.s003.docx]

**S2 Table.** Profile of 393 bacteria isolated from blood culture in neonatal sepsis.

| **Gram-negative bacteria** | **127 (32.3%)** |
| --- | --- |
| *Escherichia coli* | 27 (6.7%) |
| *Klebsiella pneumoniae* | 27 (6.7%) |
| *Acinetobacter baumannii* | 15 (3.7%) |
| *Enterobacter cloacae* | 11 (2.7%) |
| *Elizabethkingia meningoseptica* | 6 (1.5%) |
| *Pandoraea sputorum* | 5 (1.2%) |
| *Stenotrophomonas maltophilia* | 5 (1.2%) |
| *Salmonella* species | 4 (1.0%) |
| *Burkholderia cepacia* | 3 (0.7%) |
| *Achromobacter xylosoxidans* | 3 (0.7%) |
| *Pseudomonas aeruginosa* | 3 (0.7%) |
| *Pantoea* species | 3 (0.7%) |
| *Serratia marcescens* | 3 (0.7%) |
| *Sphingomonas paucimobilis* | 2 (0.5%) |
| *Proteus vulgaris* | 2 (0.5%) |
| *Acinetobacter schindleri* | 1 (0.2%) |
| *Cronobacter sakazaki* | 1 (0.2%) |
| *Enterobacter asburiae* | 1 (0.2%) |
| *Enterobacter gergoviae* | 1 (0.2%) |
| *Enterobacter kobei* | 1 (0.2%) |
| *Klebsiella variicola* | 1 (0.2%) |
| *Pseudomonas fluorescens* | 1 (0.2%) |
| *Pseudomonas stutzeri* | 1 (0.2%) |
| **Gram-positive bacteria** | **266 (67.7%)** |
| *Staphylococcus aureus* | 18 (4.4%) |
| *Streptococcus mitis* | 4 (1.0%) |
| *Enterococcus faecalis* | 3 (0.7%) |
| *Enterococcus faecium* | 3 (0.7%) |
| *Streptococcus pyogenes* | 3 (0.7%) |
| *Streptococcus agalactiae* | 2 (0.5%) |
| *Streptococcus gallolyticus* | 1 (0.2%) |
| *Staphylococcus epidermidis* | 134 (33.1%) |
| *Staphylococcus haemolyticus* | 41 (10.1%) |
| *Staphylococcus hominis* | 27 (6.7%) |
| *Staphylococcus warneri* | 14 (3.5%) |
| *Staphylococcus capitis* | 11 (2.7%) |
| *Staphylococcus saprophyticus* | 2 (0.5%) |
| *Staphylococcus kloosii* | 1 (0.2%) |
| *Staphylococcus lugdunensis* | 1 (0.2%) |
| *Staphylococcus pasteuri* | 1 (0.2%) |
