# Supplementary material for: Clinical and laboratory factors associated with neonatal sepsis mortality at a major Vietnamese children’s hospital
Source: PLOS Glob Public Health. 2022 Sep 2;2(9):e0000875. doi: 10.1371/journal.pgph.0000875 (PMC10021837; doi:10.1371/journal.pgph.0000875)
Supplement: Attachment — Submitted filename: Sepsis_050422_SGB_CL.docx [file pgph.0000875.s004.docx]

Professor Stephen Baker

Head of Enteric Infections

sbaker@oucru.org

2^nd^ September 2021

Re: Submission to PLoS Global Public Health

Dear Editor,

On behalf of all authors, I wish the article entitled “**Clinical and laboratory factors associated with mortality in neonatal sepsis patients at a major children’s hospital in Vietnam**” by Nguyen Duc Toan and other authors to be considered for publication as a research article in your journal. We thank the reviewers and editor for their comments, we have addressed them in the modified manuscript and outline them in turn below.

Reviewer #1: Abstract:
Line 44: Denominator for positive cultures is 405. Based the number of subjects and the number of positive cultures, this number should be 524 or 344, respectively. Correct or explain where 405 comes from.

- This has been added in the abstract and the results

Methods:
line 122: Since the goal of the paper is to identify factors predictive of mortality, it is unclear why the patients at highest risk of death were excluded. Please explain.

- This is now described in the methods “Patients were excluded if informed consent was not provided, if the length of hospital stay was <24 hours, or if death was predicted within 12 hours; these patients were excluded as samples and data were difficult to collect within this time during palliative care and was deemed unethical.”

line 130: How was a "presumptive pathogen" defined? And elsewhere, how were contaminants defined? There are several organisms included in Table S2 that I would have classified as likely contaminants. Please explain how the distinction between presumptive pathogens and contaminants was made with special attention given to organism that were considered to be pathogens that are typically considered to be contaminants.

- A presumptive pathogen is a non-contaminant, this has been defined in the methods. A contaminant/non-contaminant was determined by local guidelines and interpreted by the internal microbiology laboratory and confirmed by the collaborating research laboratory, this now been defined in the methods.

line 173 MALDI-TOF is a very expensive piece of equipment. Although Vietnam is classified as a LMIC, the hospital in this paper was obviously well resourced. Babies had access to parenteral nutrition, advanced diagnostics, etc. This is reflected in the high proportion of ELBWs. Their findings will not be generalizeable to most LMIC settings.

- Agreed. The organisms were identified on site and then latterly confirmed in a research lab with a Bruker system, this has been confirmed

Results:
line 204: how were contaminants defined?

- According to local guidelines, this has been added

line 206: change 34 to 344

- This has been changed

line 213: change "mortality was recorded for" to "death occurred in"

- This has been changed

line 225: add comma after isolated for readability

- This has been changed

line 226: add "commonly" after most

- This has been changed

line 228: Reference supplemental table 2

- This has been changed

line 258: For the laboratory abnormalities associated with mortality, when are they predictive? Ever? At diagnosis? Another timepoint?

- At the time of diagnosis, this has been added

lines 260-265: don't report odds ratios and confidence intervals here since they are in the table

- These have been removed

Discussion:
line 331: Add to limitations sections discussion about this hospital being a high-resource hospital.

- This has been added with context

line 342: Unclear what this sentence means. Would change to indicate that that these factors are associated with mortality in this study.

- This has been edited

Reviewer #2: What positively stands out?
This Manuscript presents a scientifically sound topic of significant global public health interest. The Author makes a good case for why this study is important. The title is very specific and does not assume or bias the reader. The Author makes effort to explain the methods used in scientifically sound terms, while avoiding jargon. The methods used are explained in simple terms.

- Many thanks for these comments

-It would be great to have a trial profile for this study, so as to follow through how patients were screened and enrolled, with a snapshot of reasons for exclusion.

-Participants who were excluded if death was 'predicted' within the subsequent 12 hours creates investigator selection bias which should be declared under limitations as it impacts on the true picture of neonatal mortality at this facility.

- An explanation has been added to the methods and in the limitations

-38.5% of the neonates (study participants) were born prematurely and hence the low birth weight; which also had an impact on poor outcomes. This figure is quite significant and shouldn't be brushed aside. This limits the generalisability of the findings to all neonates. This limitation should be acknowledged as the clinical (and underlying immunological factors and responses to disease) factors for full term neonates may way be very different.

- This has been added to the limitations

-It's great that clarification was made that selection of the 7 variables to consider in testing for associations was made on clinical judgement; however, most of them were skewed towards the laboratory picture

- Yes this is correct

-With a high blood culture positivity of 65.6%; it raises the question of what would the impact of treatment received on outcomes

- This would be largely dependent on antimicrobial susceptibility and time to treatment

-The recommendations for I.V Immunoglobulins, exchange blood transfusions and parenteral hydrocortisone are very intelligent measures but not practical or for LMIC settings. The global public health issue here would be to focus more on how to prevent the 'Pre-sclerema' stage, since it's presence is already an indicator of poor prognosis; despite the interventions

- Agreed, this has been added to the discussion

Our work identified key patient and clinical characteristics affecting mortality from neonatal sepsis in our setting. These data will enable early risk stratification and interventions appropriate to LMIC settings, in addition to guiding the training and education of healthcare professionals in developing more of a risk-based approach to patient care including the importance of infection prevention and control measures. We think these data are interesting, concerning, highly topical and likely to be of significant interest to your readership.

Yours sincerely,

**Stephen Baker BSc DIC PhD**

Wellcome Trust Senior Research Fellow

Director of Research for Global Health
