# Supplementary material for: Clinical and laboratory factors associated with neonatal sepsis mortality at a major Vietnamese children’s hospital
Source: PLOS Glob Public Health. 2022 Sep 2;2(9):e0000875. doi: 10.1371/journal.pgph.0000875 (PMC10021837; doi:10.1371/journal.pgph.0000875)
Supplement: Attachment — Submitted filename: Sepsis_060622_SGB_CL.docx [file pgph.0000875.s005.docx]

Professor Stephen Baker

Head of Enteric Infections

sbaker@oucru.org

6^th^ June 2022

Re: Submission to PLoS Global Public Health

Dear Editor,

On behalf of all authors, I wish the article entitled “**Clinical and laboratory factors associated with mortality in neonatal sepsis patients at a major children’s hospital in Vietnam**” by Nguyen Duc Toan and other authors to be considered for publication as a research article in your journal. We have addressed the final comment in the manuscript, this is described below.

Reviewer #1: Methods section: (no page or line numbers were included in the resubmission)
Under study definitions, 1st paragraph, last line: presumptive pathogens are, by definition, non-contaminants. What remains to be clarified is how contaminant vs. presumptive pathogen was defined. The following paragraph states that they were "confirmed using local guidelines" and the results section says they "were determined to be contaminants by local clinical microbiology guidelines". Please provide the guidelines in the methods section. There are many organisms included in Supplemental table 2 which are nearly always contaminants. How were the 4 micrococcus cultures deemed to be pathogenic? For the >100 babies with CoNS, how were these defined to be pathogenic? Others that need justification for their inclusion: Cupriavidus, Halomonas, Ochrobactrum, Paenibacillus, Ralstonia, Roseomonas, Rothia, Kytococcus. These organisms have only rarely been considered to be a human pathogens and are environmental, skin or gut commensals. How do the "local clinical microbiology guidelines" handle these organisms?

This definition has been glossed over by the authors but is vital to the relevance of their paper. If a quarter of the patients they include do not have a plausible putative pathogen identified, they have included many patients who don't truly meet the inclusion criteria and really calls into question the validity of their results.

If they cannot justify why typical contaminants were considered pathogenic in these patients, they should consider removing these patient cases altogether or, at minimum, provide a sensitivity analysis with these cases removed.

- We appreciate your effort with this issue, this was a constant source of discussion during the protocol development. The protocol states “Organisms including coryneforms (Corynebacterium, etc), Micrococci, Propionibacterium, Bacillus, alpha haemolytic Streptococci, environmental Gram-negative bacilli and non-pathogenic Neisseria will be considered potential contaminants. The pathogen-contaminant decision will be made based on the clinical relevance of the isolated bacteria and the independent assessments by two qualified medical microbiologists. If there is disagreement, then the case will be discussed until a decision is reached”. This definition has been added to the methods. We reviewed the organisms in supp. Table 2 and removed those that meet these criteria and the additional contaminants to better meet the protocol definition and the recommendations of the reviewer. Coagulase negative staph is a common skin contaminant but after discussion with the resident microbiologists was kept as a pathogen, given that Coagulase negative staph is a common cause of sepsis in vulnerable groups, such as neonates. Additionally, the reviewer states “they have included many patients who don't truly meet the inclusion criteria and really calls into question the validity of their results”. This is incorrect as the recruitment criteria were “ This study included all in-patient neonates (≤28 days of age) at CH1 clinically diagnosed with a primary probable sepsis and receiving a blood culture from January 2017 to June 2018. This center does not provide obstetrical care; therefore, the study participants were out-born neonates. Patients were excluded if informed consent was not provided, if the length of hospital stay was <24 hours, or if death was predicted within 12 hours; these patients were excluded as samples and data were difficult to collect within this time during palliative care and was deemed unethical.” Therefore, this would not change the number of being recruited and would only change the proportion with culture confirmed sepsis. Notably, the majority of risk factors were clinical and non were microbiological.

Our work identified key patient and clinical characteristics affecting mortality from neonatal sepsis in our setting. These data will enable early risk stratification and interventions appropriate to LMIC settings, in addition to guiding the training and education of healthcare professionals in developing more of a risk-based approach to patient care including the importance of infection prevention and control measures. We think these data are interesting, concerning, highly topical and likely to be of significant interest to your readership.

Yours sincerely,

**Stephen Baker BSc DIC PhD**

Wellcome Trust Senior Research Fellow

Director of Research for Global Health
